# User involvement and value co-creation in well-being ecosystems

**DOI:** 10.1108/JHOM-11-2022-0339

**Published:** 2023-09-26

**Authors:** León Poblete, Erik Eriksson, Andreas Hellström, Russ Glennon

**Affiliations:** Department of Business Studies, University of Uppsala , Uppsala, Sweden; Department of Technology Management and Economics , Chalmers University of Technology , Gothenburg, Sweden; Department of Strategy , Enterprise and Sustainability , MMU Business School , Manchester Metropolitan University , Manchester, UK; The University of Sydney Business School , Sydney, Australia

**Keywords:** Well-being ecosystems, Value co-creation, User involvement, Resource reconfiguration, Social innovation

## Abstract

**Purpose:**

This article aims to examine how users' involvement in value co-creation influences the development and orchestration of well-being ecosystems to help tackle complex societal challenges. This research contributes to the public management literature and answers recent calls to investigate novel public service governances by discussing users' involvement and value co-creation for novel well-being solutions.

**Design/methodology/approach:**

The authors empirically explore this phenomenon through a case study of a complex ecosystem addressing increased well-being, focussing on the formative evaluation stage of a longitudinal evaluation of Sweden's first support centre for people affected by cancer. Following an abductive reasoning and action research approach, the authors critically discuss the potential of user involvement for the development of well-being ecosystems and outline preconditions for the success of such approaches.

**Findings:**

The empirical results indicate that resource reconfiguration of multi-actor collaborations provides a platform for value co-creation, innovative health services and availability of resources. Common themes include the need for multi-actor collaborations to reconfigure heterogeneous resources; actors' adaptive change capabilities; the role of governance mechanisms to align the diverse well-being ecosystem components, and the engagement of essential actors.

**Research limitations/implications:**

Although using a longitudinal case study approach has revealed stimulating insights, additional data collection, multiple cases and quantitative studies are prompted. Also, the authors focus on one country but the characteristics of users' involvement for value co-creation in innovative well-being ecosystems might vary between countries.

**Practical implications:**

The findings of this study demonstrate the value of cancer-affected individuals, with “lived experiences”, acting as sources for social innovation, and drivers of well-being ecosystem development. The findings also suggest that participating actors in the ecosystem should utilise wider knowledge and experience to tackle complex societal challenges associated with well-being.

**Social implications:**

Policymakers should encourage the formation of well-being ecosystems with diverse actors and resources that can help patients navigate health challenges. The findings especially show the potential of starting from the user's needs and life situation when the ambition is to integrate and innovate in fragmented systems.

**Originality/value:**

The proposed model proposes that having a user-led focus on innovating new solutions can play an important role in the development of well-being ecosystems.

## Introduction

Ageing populations have increased the number of people living with chronic and/or multiple diseases in many Western countries ( Eriksson *et al.* , 2020 ) and many healthcare systems have not kept up with this progress ( Mintzberg, 2017 ; Porter and Teisberg, 2006 ). Since the 1980s, the predominant ways of improving healthcare management and systems (e.g. not clinical advances) have been drawn from private-sector manufacturing, often labelled New Public Management (NPM) (e.g. Osborne *et al.* , 2013 ). The goods logic of manufacturing has been criticised for leading to “producing” public services and promoting a linear, assembly line-like approach in which a public organisation's focus is inward-looking to internal processes ( Osborne, 2018 ). As a result, different actors work in silos, increasing the risk of service users falling through the cracks in such fragmented systems ( Eriksson *et al.* , 2020 ). Therefore, NPM is often claimed to be unfit to address complex challenges in contemporary societies, such as ageing populations, pandemics and forced migration ( Christensen and Lægreid, 2011 ).

This position is problematic mainly for two reasons. Firstly, the user's role in healthcare innovation activities is often deemed as passive, simply a receiver of services and consumer of value ( Skålén *et al.* , 2018 ). Secondly, top-down producer-driven solutions do not always meet users' expectations either, because the offered alternatives are frequently too distant from what users actually need (e.g. Bergman *et al.* , 2015 ). Hence, users' involvement can help to bridge this distance because their solutions are directly built on problems related to their everyday practices and needs ( Trischler *et al.* , 2018 ). Some of the attempts to address the weaknesses of NPM have helped to generate a focus on Public Service Logic (PSL) ( Osborne, 2018 , 2020 ), which seeks to highlight the different ways that public and private services operate in theory and practice and foregrounds the multiple roles of service “users” and other actors.

In this new frontier, the role of the user has changed from isolated to connected, unaware to informed and passive to active ( Eriksson and Hellström, 2021 ). Users do not passively wait for value to be delivered, but rather they are active co-creators of that value ( Osborne, 2018 ). Thus, users may co-design and co-produce products and services by contributing their time, knowledge and skills ( Grönroos, 2019 ). Accordingly, the traditional view of company-centric value creation is obsolete. However, the dominant, limited focus of analysis has been on isolated, existing services as well as the user-provider dyad; this has generated little knowledge about how value between multi-actors (individuals and organisations) in ecosystems is co-created. Thus, an ecosystem approach ( Poblete *et al.* , 2022 ; Kinder *et al.* , 2022 ; Ruijer *et al.* , 2023 ; Osborne *et al.* , 2021 ; Petrescu, 2019 ) with a multiplicity of actors is necessary to produce innovative solutions that can tackle contemporary societal challenges *,* including healthcare, health and well-being in general.

Many healthcare services engage with numerous actors including private companies, governmental organisations, NGOs, patients, families and healthcare professionals ( Eriksson *et al.* , 2020 ). As the participation of all these actors is essential for exceptional care, new approaches are changing the organisation of the healthcare arena, including the flows of medical knowledge, clinical information and the availability of resources ( McColl-Kennedy *et al.* , 2012 ). Hence, more cooperation and coordination among healthcare organisations and other actors is necessary. This is distinct from many of the existing network ( Kinder *et al.* , 2022 ; Nenonen and Storbacka, 2010 ) or collaboration models ( Agranoff and McGuire, 2003 ; Provan and Kenis, 2008 ); the ecosystem view puts the experience of the users at the heart of value creation (e.g. Adner, 2006 ). As Jacobides *et al.* (2018) put it, ecosystems refer to “the collaborative arrangements through which firms combine their offerings into a coherent, customer-facing solution”. This allows for the exchange and development of diverse resources ( Prenkert *et al.* , 2022 ), enabling groups of actors to deliver integrated and innovative solutions to end-users ( Clarysse *et al.* , 2014 ; Jacobides *et al.* , 2018 ). Kinder *et al.* (2022) also emphasise that public services require a new analytical framework based on ecosystems. Whilst PSL certainly helps to consolidate some of these aspects of service orientation, more work is needed to answer calls for PSL to adequately explore (amongst other issues) how multiple actors act, interact and react in the provision of complex public services ( Osborne *et al.* , 2022 ).

Even though users can be active participants in exchanges (e.g. Osborne, 2018 ), and ecosystems are increasingly regarded as important vehicles to create and capture value from complex value propositions ( Dattée *et al.* , 2018 ), research on users' involvement and multi-actor value co-creation in ecosystems to tackle complex societal challenges remains rather scarce. In this paper, we take inspiration from work by Dattée *et al.* (2018) , Adner (2006) , Jacobides *et al.* (2018) and Kinder *et al.* (2022) , to develop new theoretical insights into the interplay between users' involvement, value co-creation and ecosystem structures to address well-being challenges. The purpose of this paper, therefore, is to contribute to the public management literature and answer recent calls to investigate novel public service governances and logics ( Kinder *et al.* , 2022 ; Osborne *et al.* , 2021 ; Osborne, 2020 ) by discussing users' involvement and value co-creation for novel well-being solutions. More specifically, we aim to answer the two following interconnected research questions:

RQ1.
What are the characteristics of users' involvement for value co-creation in the development of innovative, well-being ecosystems?

RQ2.
How does users' involvement in value co-creation influence the orchestration of innovative, well-being ecosystems?

Creation is used as an overarching concept in the paper, where different actors engage in activities with the common goal of creating new concrete services, in this case HoP. Development is used to describe the progression and joint efforts/activities to drive the design process forward. Finally, orchestration is used to explain how the development and creation of the service itself is managed. How resources are integrated and combined with the effort to create value at different levels in the system; how arenas, activities and processes for actors to act and interact are managed.

The research questions are of particular interest to researchers as well as policymakers, who are increasingly investing in innovation to foster these types of ecosystems. We provide novel insights about ecosystems as structures enabling users' involvement and value co-creation in the development and orchestration of a real-life well-being ecosystem, the House of Power (HoP) *,* Sweden's first patient-led support centre for people affected by cancer. We draw on longitudinal action research to develop a richer understanding of user involvement by focussing on the formative evaluation stage of a longitudinal evaluation of HoP.

Our findings contribute to the public management literature in several respects. We find that resource reconfiguration of multi-actor collaborations provides a platform for unique value co-creation, innovative health services and powerful user involvement. Common themes we developed include the engagement of essential actors in the ecosystem, the need for multi-actor collaborations to reconfigure heterogeneous resources, actors' adaptive change capabilities and the role of governance to align the diverse ecosystem components. Further, our findings show that users' involvement in value co-creation influences the development and orchestration of ecosystems, which directly contributes to tackling complex societal well-being challenges. Moreover, the elaboration of the value concept in a public sector context may contribute to value in ecosystems literature.

The paper unfolds as follows. First, we provide a literature review with a brief discussion of concepts related to ecosystems, user involvement, resource reconfiguration and value co-creation. We subsequently describe the method we used to collect and analyse the data. Finally, we discuss the empirical results, along with related implications for practitioners and policymakers and future trends.

## Theoretical background

### Ecosystems in healthcare and public management

Ecosystem is an increasingly influential concept in public management ( Kinder *et al.* , 2022 ; Osborne *et al.* , 2021 ; Ruijer *et al.* , 2023 ; Petrescu, 2019 ) and organisational theory ( Jacobides *et al.* , 2018 ; Adner, 2017 ). The concept relates to a group of interacting actors that depend on each other's activities ( Ganco *et al.* , 2020 ; Jacobides *et al.* , 2018 ; Moore; 2006 ; Poblete *et al.* , 2022 ). While Adner (2017 , p. 42) refers to the ecosystem as the alignment structure of the multilateral set of partners that need to interact for a focal value proposition to materialise, Jacobides *et al.* (2018) define ecosystems as “the collaborative arrangements through which firms combine their offerings into a coherent, customer-facing solution”. Although ecosystem relationships do not require formal alliances ( Poblete *et al.* , 2022 ), and do not bind organisations, links between organisations express their co-dependence brought about by their mutual co-specialisation (e.g. Alexy *et al.* , 2013 ; Kapoor and Lee, 2013 ). Ecosystems, thus, reflect interdependencies between organisational entities, be they directly connected or indirectly related ( Jacobides *et al.* , 2018 ; Kapoor, 2018 ). According to Bryson *et al.* (2022) , actors in an ecosystem can obtain a sharper understanding of their strengths, weaknesses, opportunities and threats, as well as competitive and collaborative capabilities and advantages.

In this regard, health can be regarded as an ecosystem of multiple actors that is more complex than the basic view that patients passively receive care from, say, hospitals or doctors ( Eriksson and Hellström, 2021 ). Patients are increasingly viewed as active contributors to their own healthcare outcomes, and there is growing evidence that supports the benefit of a user-centred approach to healthcare ( Porter and Lee, 2013 ). Such an approach demands healthcare be designed around the specific needs of a user.

Another important aspect is the incorporation of a broad range of actors and a wide range of collaborative activities into the design of these ecosystems ( Michie *et al.* , 2003 ). For example, a team-based approach of shared decision-making between medical specialists, nursing staff, the patient and their family encourages a holistic approach to patient care ( Barry and Edgman-Levitan, 2012 ). Bryson *et al.* (2022) also emphasise that an ecosystem perspective on health issues outlines as significant social sustainability, personalised interactions, sociability, support and reciprocity, as well as the potential for an interdisciplinary approach to public service. While Petrescu (2019) argues that the ecosystem can be a framework through which to understand the complexities of public service delivery and value creation at the societal, service and individual levels, Kinder *et al.* (2022) and Osborne *et al.* (2021) stress that ecosystems represent the most convincing framework for understanding public service delivery. This involves a greater understanding of an ecosystem and the multiple actors within it ( Bryson *et al.* , 2022 ) to evaluate the development of well-being ecosystems to help tackle complex societal challenges. However, despite these new perspectives, little research has been undertaken on the dynamics of a well-being ecosystem from the viewpoint of multiple participants, focussing on users' involvement and their collaborative practices, i.e. not clinical practice or behaviours.

### Value co-creation in ecosystems

Historically, “value” referred to the value produced through the manufacture and distribution of tangible goods ( Prahalad and Ramaswamy, 2004 ; Vargo *et al.* , 2008 ). However, recent years have witnessed a growing tendency towards a more systemic view of value co-creation ( Vargo and Akaka, 2012 ). Galvagno and Dalli (2014 , p. 644) define value co-creation as “the joint, collaborative, concurrent, peer-like process of producing new value, both materially and symbolically.” Thus, value creation in an ecosystem is enabled by complementarities and interdependencies between actors, which contributes to the user value proposition ( Kapoor and Agarwal, 2017 ; Kapoor, 2018 ).

Value creation has shifted from a goods-dominant logic, where tangible goods are created within an organisation, to a joint process where value is co-created in an ecosystem, based on a service-dominant logic (S-D logic) ( Vargo *et al.* , 2008 ; Vargo and Lusch, 2004 ). S-D logic focuses on the exchange of services during which one actor uses a set of skills and capabilities to benefit another actor. In value co-creation, value is not located in products and services themselves but rather in usage and experience ( Vargo and Lusch, 2008 ). Prahalad and Ramaswamy (2004) proposed a new frame of reference for value co-creation, noting that: “The use of interactions as a basis for co-creation is at the crux of our emerging reality.” Their starting premise (p. 15) was that “value is co-created”, with two additional premises of “co-creation experiences are the basis of value” and “the individual is central to the co-creation experience”. Osborne (2020) and Petrescu (2019) point out that the concept of ecosystem, focused on integrating actors and resources, can be beneficial in drawing new conceptual avenues for value co-creation in public services.

### Resource reconfiguration to create value in ecosystems

Actors within an ecosystem are attracted to share their resources, responding to value propositions that offer the potential for mutually beneficial outcomes ( Davis, 2016 ). An ecosystem is dynamic as resources are employed and shared between the actors, thus altering their availability and the attractiveness of respective offerings ( Frow *et al.* , 2015 ). According to Vargo and Lusch (2016 , p. 161), ecosystems include “a system of resource-integrating actors connected by shared institutional arrangements and mutual value creation through service exchange”. Thus, resource reconfiguration can be defined as the modification of a resource as an attempt by an actor to obtain benefits in response to environmental changes ( Chou and Zolkiewski, 2012 ; Karim, 2006 ; Poblete, 2021 ). Consequently, resource reconfiguration is characterised by the adjustment and reorientation of a resource ( Galunic and Rodan, 1998 ; Prenkert *et al.* , 2022 ) to co-create new value in ecosystems.

### User involvement in ecosystems

As shown, the service-dominant logic approach (e.g. Grönroos, 2011 ; Vargo and Lusch, 2004 ) emphasises the importance of the micro-level ( Hardyman *et al.* , 2015 ; Olsson, 2016 ) and the relational and interactional aspects between customer and provider in value co-creation ( Grönroos, 2011 ; Tronvoll, 2012 ). Healthcare provision has historically been regarded as a process through which patients passively receive care from professionals ( Berwick, 2009 ), ignoring patients as increasingly active contributors to their healthcare outcomes ( Bergman *et al.* , 2015 ). There is growing evidence that supports the benefits of user involvement in health ( Porter and Lee, 2013 ). Users are active ( Osborne *et al.* , 2013 ), participating not only in the co-production of innovative solutions, but also in co-designing solutions by contributing their experiences and expectations ( Osborne *et al.* , 2016 ; Trischler *et al.* , 2019 ).

Conceptually, two aspects of user involvement in ecosystems are germane. First, organisations need to know which users are capable of providing valuable inputs ( Gruner and Homburg, 2000 ; Von Hippel, 1986 ). This dimension contains knowledge about critical user characteristics. Hence, an important aspect of this approach is the incorporation of users as crucial contributors. Second, users require a wide range of collaborative activities to facilitate the co-design of healthcare ( Michie *et al.* , 2003 ).

### Implications for the research focus

This paper focuses on users' involvement for value co-creation in the development of innovative well-being ecosystems to help tackle societal challenges. Wellbeing ecosystems must engage both organisations and users in interactions to co-create value. That is, while organisations can understand users' needs and provide customised services and solutions, users can provide valuable information and ideas to those organisations. Thus, we define well-being ecosystems as structures of interaction and exchange among participating actors that facilitate resource reconfiguration and value co-creation, driven by user-led solutions.

## Methodology

### Research strategy

We sought to deepen our understanding of the characteristics of user involvement for value co-creation in the development and orchestration of innovative ecosystems to tackle well-being challenges; consequently, we chose an exploratory, qualitative research design ( Eisenhardt, 1989 ; Yin, 2018 ) arising from an in-depth case study ( Langley *et al.* , 2013 ; Silverman, 2015 ; Yin, 2018 ). Because of the explorative approach and the novelty of a service such as HoP in a Swedish context, a multiplicity of data collections was conducted ( Fusch and Ness, 2015 ), which is a well-established tradition in the social sciences ( Alexander *et al* ., 2008 ). Moreover, to get as many stakeholders' perceptions into the project as possible we collected individual interviews (e.g. when there was a risk that more sensitive issues could be discussed), focus groups (e.g. when participants new each other well) and so forth. Case study research is widely accepted as an effective way to understand and explain complex inter-organisational relationships to develop theoretical insights (e.g. Eisenhardt, 1989 ). This enabled a deeper understanding of the “black box” of the characteristics of users' involvement in value co-creation in the development and orchestration of innovative, well-being ecosystems.

A single case study was deemed relevant as these can function as a critical case ( Yin, 2018 ) and as such constitute powerful examples rather than representative samples ( Siggelkow, 2007 ). The HoP case is based on an ongoing longitudinal action research project ( Lifvergren *et al.* , 2015 ; Bradbury, 2010 ; Brydon-Miller *et al.* , 2003 ) and draws on established traditions within the management discipline ( Coghlan and Brannick, 2014 ). It started in 2016 in which one of the authors participated as an inside action researcher throughout the project. The account presented in this paper reflects one of the many possible theoretical trajectories that was identified during the effort of abduction and systematic combining ( Locke *et al.* , 2008 ; Dubois and Gadde, 2002 ).

### Data collection

We compiled data from a wide range of sources. The data collection process included 21 semi-structured interviews ( Table 1 ), a workshop series, observations, a focus group study and secondary material. All interviews, except for one, were recorded and transcribed verbatim and extensive field notes were taken during every interview to rectify any possible misunderstanding later on ( Tranfield *et al.* , 2003 ). The recording of one interview failed due to technical reasons and notes were taken instead. One of the authors acted as an inside action researcher conducting interviews with actors in the ecosystem – the primary data source in this paper. The interviewer also facilitated a focus group with eight visitors to HoP. Both the interviews and focus groups addressed questions such as: How were you involved in developing House of Power? What kind of resources could you contribute with in the design process? How was collaboration with other stakeholders? Did you miss any particular stakeholder in the design process? What was your role in developing HoP? What knowledge, skills, experiences, etc. could you contribute with during the development process? How did you perceive the development/design process? How was the interaction with the other actors in the design process? What does HoP contribute with?

Participant observation by one of the authors, who was directly involved in the first and second phases of the development also informed the study. The observation protocol also served as a way for the team to reflect during and after the various sessions and included, e.g. group dynamics, sense of progress and direction of the development, suitability of working methods. One of the researchers asked questions and another observed interactions between focus group participants. The observer noted no particular conflicts, and the participants overall tended to agree with one another, seeking consensus.

Data were gathered at workshops (between 9 and 51 participants) ( Brydon- Miller *et al.* , 2003 ). Beyond researchers' field notes, qualitative data was also gathered via focus groups, observations, emotional mapping ( Donetto *et al.* , 2015 ), design workshops, dialogue meetings ( Huzzard *et al.* , 2017 ), business model canvas workshops ( Osterwalder and Pigneur, 2010 ) and interviews with cancer patients. We have also referenced unpublished memos and presentations, particularly around the evolution of the ecosystem.

We employed several strategies to assess the quality of the findings. First, we gauged the research's overall rigour according to the criteria for credibility and trustworthiness ( Lincoln and Guba, 1985 ). We interviewed a range of actors participating in the ecosystem because informants with different perspectives reduce bias by triangulating perceptions of phenomena ( Golafshani, 2003 ). We also employed the so-called *logical coherence* ( Dubois and Gadde, 2002 ) — that is, the appropriate matching of reality and theoretical constructs — to reinforce the study's validity and provide a rich set of quotations from interviews to illustrate and support key findings ( Corbin and Strauss, 2014 ). Further, using secondary sources and multiple interviews within the same actors ( Bell *et al.* , 2018 ; Yin, 2018 ) contributed to the triangulation of the empirical material, captured other dimensions of the phenomenon and enhanced the credibility of the results ( Golafshani, 2003 ). In turn, by indicating the transferability of the findings for use in other empirical settings, triangulation assured the rigour of the study and the credibility of our findings ( Zeithaml *et al.* , 2020 ).

### Description of the empirical context

The study reported here had no *a priori* research plan; the design emerged iteratively and progressively. This progressive focussing is detailed here to remain authentic to the non-linearity and unpredictability of our research process ( Sinkovics and Alfoldi, 2012 ). The HoP is the first cancer support centre in Sweden initiated and led by cancer-affected people. It is a social innovation that uses the lived experience of cancer survivors as a basis for identifying gaps in the welfare system. It is managed as a non-profit organisation with a business model that integrates relevant resources in society in new ways. The needs of the cancer-affected have also been translated into the spatial design of a 300 square metres venue.

One in three people in Sweden will be diagnosed with cancer during their lifetime, and almost 40% of these are children or people of working age. Meanwhile, better treatment and earlier detection mean that more people are living longer with the disease ( Socialstyrelsen and Cancerfonden, 2013 ). A cancer diagnosis affects a person physically, mentally and socially. Returning to a well-functioning life after cancer requires rehabilitation and cooperation between many different organisations. Cancer patients and relatives often find that the available psychosocial support during and after treatment is insufficient ( Olsson, 2016 ). Without adequate support, finding your new identity and way back to everyday life is difficult. In addition, the risk of relatives becoming sick due to stress caused by the illness increases by 25% one year after diagnosis ( Sjövall *et al.* , 2011 ). Consequently, there is a need to rethink how society's resources can be better integrated and reconfigured to support people directly and indirectly affected by cancer.

### Data analysis

We followed an abductive approach to data analysis and theory building ( Locke *et al.* , 2008 ), which involved (1) applying an established theory, (2) observing a surprise in the empirical phenomenon in light of the theory and (3) articulating a new theory that resolves the surprise ( Alvesson and Karreman, 2007 ). We fulfilled these steps iteratively by moving back and forth between data and theory ( Dubois and Gadde, 2002 ). Our analysis started by reading and re-reading the empirical material and organising the events into progressively coherent narratives. Here the interviews served as a base, but other data from the workshop and fieldwork supplemented and strengthened the analysis and the creation of the thematic structure. Our different researcher roles and data sources also helped to generate a creative dialogue about both parts and wholes of the case studied and its ecosystem.

**Figure 1:**
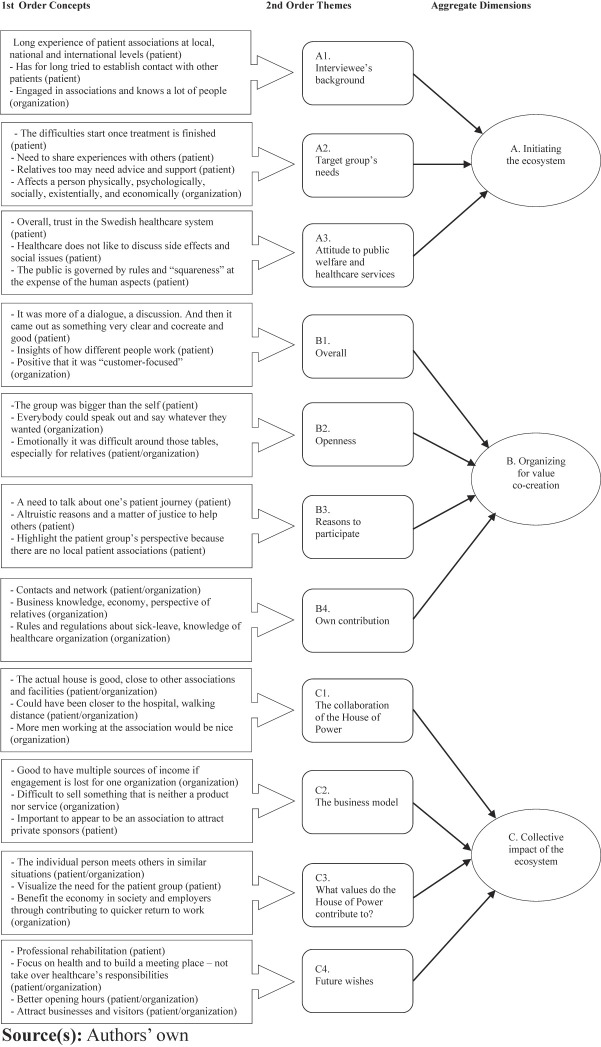
Coding structure

The transcriptions were colour-coded based on similarities/differences and sorted into first order concepts (see Figure 1 ). Thus, the empirical material was sorted into first-order concepts in which we stayed close to the respondents' answers, such as used expressions and vocabulary.

Based on similarities between these first-order concepts, second-order themes were constructed ( Gioia *et al.* , 2013 ), leading in turn to overarching aggregate dimensions. As seen in Figure 1 , the HoP case exhibited three distinct, evolutionary phases, which we called: (1) Initiating the ecosystem, (2) Organising for value co-creation and (3) Collective impact of the ecosystem respectively.

## Findings

As discussed in the conceptual framework, the interplay between users' involvement, value co-creation and ecosystem structures is crucial for creating innovative well-being solutions. Knowledge has, thus, been generated by researchers and practitioners together. In these contexts, praxis has also been referred to as actionable knowledge ( Argyris, 2004 ); that is, knowledge that is local and is of benefit to the participants. The case is structured chronologically in three phases in the evolution of the ecosystem: (1) initiating the ecosystem, (2) organising for value co-creation in the ecosystem and (3) the collective impact of the ecosystem [1] .

1. Initiating the ecosystem January 2016 to April 2016

The initiative that became HoP was started by the Patient and Relatives Council (PRC) at the Regional Cancer Center West (RCC West). One of the highest priorities for the PRC was the shortcomings in psychosocial support in cancer care. In this project's pre-study phase, many of the patients and relatives expressed a desire to meet others in the same situation to share experiences and to create a sense of community and safety. Or in the words of a project participant: “Healthcare deals with the tumour, but what about the rest? What about the life?” Thus, an important goal for the HoP was to provide social, emotional and practical support that complemented the traditional healthcare offer. This expands the scope beyond the traditional healthcare sphere, which is a challenge for the welfare system. A project team was put together with complementary knowledge and skills of cancer care, regional healthcare systems, rehabilitation, psychosocial support, innovation, improvement and design methodologies. One of the project members also had first-hand experience from being a cancer patient and was one of the key individuals initiating this exploration.

- Organising for value co-creation in the ecosystem: May 2016 to December 2016

This phase focused on values and principles needed for creative and innovative exploration. Again, there was no detailed and pre-planned map. Rather, the project set up some guiding principles to act as a compass; this included adopting a “life event” perspective. This perspective (i.e. receiving a cancer diagnosis) allowed the project to see the situation of the patient as a whole, thus foregrounding the individual's needs and journey through the system. This is also the natural starting point for the person affected by cancer. By recognising the complexity of the whole system, a lack of coordination between actors was revealed and new actors were identified and invited into the collaboration.

The life event perspective illustrated the involvement of multiple actors and service providers in a fragmented “system”. All relevant actors were invited with the motto of “nobody can do everything, but everybody can do something” to emphasise a no-blame and inclusive approach. Consequently, a broad range of actors was invited to the project: patients, relatives, the hospital staff, primary caregivers, the municipality members of the city of Borås, Social insurance agency staff, employment agency staff, politicians, local business owners and members of the civil society. Workshops were thus with “the whole system in the room” ( Huzzard *et al.* , 2017 ). The project used a design thinking process inspired by the Double Diamond ( Brown, 2009 ) that shifts between general and specific to capture a richness of original ideas that may then become concrete.

### Connecting participants and identifying needs

As a result of this first workshop, it was observed that the participants started to interact with each other and became acquainted. As a next step, focus groups were also held with three of the more significant institutional actors (local hospital, the Social Insurance Agency and the Employment Agency) to secure their willingness to collaborate, better understand their institutional views and identify potential resources that could be integrated within the HoP. These institutional views are captured in Table 2 .

### Visual methodologies

The next stage shifted from an institutional lens to a more individual approach to uncovering the underlying needs of cancer patients themselves through visually mapping the emotions inhabited throughout a “patient journey”. This visual methodology has been shown to have potential in exploring and processing emotions in people with cancer ( Ennis *et al.* , 2018 ). Next, the participants created a collage of images representing the joint vision of HoP. Through using images rather than words, the participants had a greater opportunity to associate individual perspectives to this vision and also to access deeper layers of meaning of their joint vision.

After having collected significant insights into needs, emotions and future visions, the project team helped the participants to capture some of these in a short film named *“What if?”* . This film integrated perspectives from many different actors, from patients, family members, to staff from hospitals and government agencies.

### Prototyping–translating needs to physical or spatial expression

The last step included prototyping and further development of the HoP concept. In this case, the prototypes were used to create a physical or spatial expression for the identified needs. This helped to concretise the requirements through a co-design process. This is relatively unusual in co-production cases, as more attention is often paid to the co-delivery of the experience than the co-design of a service ( Dudau *et al.* , 2019 ).

- Collective impact of the ecosystem: February 2018 to June 2020

The HoP opened on February 8th, 2018 as the first Swedish patient-led support centre for cancer-affected people. Whilst evaluation of its impact will require a continuation of the longitudinal study, the work so far has allowed some reflection and consideration of the design process. The design of the facility was based on the user-driven workshops that created commitment and co-ownership of the wider range of actors engaged in the process. “Engaging all stakeholders” is perhaps somewhat of a truism ( Mitchell *et al.* , 1997 ) in project management, but the HoP project's distinctive life event focus and methodological sophistication have allowed a higher degree of actor salience to emerge. The diversity of these actors is also reflected in the governing body.

Another area of distinctiveness to this project has been the ability to reconfigure existing resources around the co-designed facility, which is being run as a non-profit association and thus integrates financial and non-financial resources outside of the commercial or state mindsets. By offering emotional, social and practical support, HoP has developed a new role in Swedish welfare; it exists in the borderlands between cancer care and family support. The clear user involvement and the business model with shared social responsibility have been regarded by external assessors as innovative as a finalist in design awards, e.g. Gronroos (2019) for the most innovative development project in Sweden, International Service Design Award 2018 and Swedish Design Award 2018.

An evaluation by Smith *et al.* (2021) highlighted six clear types of value in HoP h: (1) Community for me and my relatives (2) New knowledge (3) Creativity (4) Exchange of experience (5) A gathering place for activities 6) Provides energy and strength. Many cancer sufferers experience a feeling of loneliness in their illness and HoP is described as a healing place, where informal conversations and community are central values of the business. This is a need that is unmet through traditional cancer care forms.

## Discussion

The objective of the study was to explore answers to two key research questions: (1) what are the characteristics of users' involvement for value co-creation in the development of innovative well-being ecosystems? and (2) how does users' involvement in value co-creation influence the orchestration of innovative well-being ecosystems? In evaluating the process, we have drawn upon insights from the ecosystem ( Adner, 2017 ; Ganco *et al.* , 2020 ; Jacobides *et al.* , 2018 ; Kinder *et al.* , 2022 ; Petrescu, 2019 ; Poblete *et al.* , 2022 ), Public Service Logic ( Osborne, 2018 ; Eriksson, 2019 ), value co-creation ( Galvagno and Dalli, 2014 ; Kapoor and Agarwal, 2017 ) and user involvement ( Porter and Lee, 2013 ; Osborne *et al.* , 2013 ) literature to theorise from a longitudinal case study. The empirical data reveal several findings that contribute to the development of an ecosystem approach in public management focussing on the concepts of users' involvement and value co-creation.

*First* , the paper highlights the importance of balancing actor-focus. Many of the “co-concepts” suffer from an overemphasis on the user ( Eriksson, 2019 ; Trischler *et al.* , 2019 ), which not only places unrealistic expectations on their shoulders but also is likely to be necessary but insufficient in addressing many of the complex societal challenges ( Christensen and Lægreid, 2011 ). Our findings propose that an ecosystem perspective ( Kinder *et al.* , 2022 ; Petrescu, 2019 ) seems to be more appropriate by offering balanced recognition to the resources of, for example, professionals, private and other actors that can be reconfigured in new ways ( Karim, 2006 ; Galunic and Rodan, 1998 ; Poblete, 2021 ).

At the same time, the ecosystem approach puts enough emphasis on the user's lifeworld to reveal gaps in the social systems from the user's perspective and does not solely focus on collaborations between organisations, which is a common weakness in many inter-organisational network models ( Kinder *et al.* , 2022 ; Poblete *et al.* , 2022 ; Provan and Kenis, 2008 ; Ruijer *et al.* , 2023 ). As our findings show, the user's experiences at the centre of collaboration can be a fruitful strategy to address value creation among a multiplicity of actors in the well-being ecosystem.

*Second* , it may help to clarify *why* – value for whom? – user involvement is carried out in the first place. Many of the co-concepts tend to become ends in themselves, which may be justified from a deliberative democracy perspective ( Fishkin, 2011 ). Our empirical data reveal that many actors participated for their own sake, but it was also clear that they also participated for the sake of other (future) patients. This more altruistic approach may be useful in that value must be provided for the newly created (non-profit) organisation – otherwise, it might cease to exist and not be able to create value for others. Indeed, the HoP itself contributed to societal value by filling a gap in the welfare system benefitting not only its visitors but also the broader citizenry.

*Third* , how participation may be carried out varies enormously. As in earlier studies ( Hendriks, 2012 ), the representatives in our empirical case came from diverse groups. One of the groups represented patients as board members at the regional cancer centre. These were rather “professionalized” and some had made international careers as patient representatives; relatively often they had a professional background in healthcare. Many of the representatives in this group were more “detached” and did not bring in their personal stories as much as others. The other group represented cancer patients in the local city and had a more mixed professional background. They talked more in terms of their own experiences, not least the emotional and social aspects of receiving a cancer diagnosis. Consequently, cancer patients regarded HoP more as a social meeting place where they could meet people like themselves, rather than a “semi-professional” healthcare institution. These findings may contribute to nuance the ongoing discussions of representativity of citizens in various co-concepts ( Eriksson, 2019 ) – what are the benefits and risks with each type?

### Theoretical contributions

This research makes several important theoretical contributions. Much of the existing research offers little guidance for in-depth exploration of more holistic and systematic opportunities to tackle complex societal challenges associated with well-being. The last decades have seen an increased critique of NPM, not least of which is that it neglects the unique characteristics of the public sector (e.g. Pollitt and Bouckaert, 2017 ). Osborne (2018) argues that inspiration from the manufacturing industry is less appropriate since public services are predominantly just that – services, not goods. In order not to repeat the public-sector blindness of NPM, we believe the uniqueness of the public sector needs to be brought in from the onset of ecosystems theorisations. Or in the words of Dudau *et al* . (2019 , p. 1583) value co-creation needs to be “disenchanted” and “contextualized to fit public service environments.”

One such aspect is the concept of value. For instance, individual value has often been the focus of private sector value co-creation, often by focussing on value realised by the end-user (e.g Anker *et al.* , 2015 ). Naturally, in an ecosystems view, the multiplicity of actors entails that all actors contribute to the joint creation of value – for themselves and others ( Eriksson *et al.* , 2020 ). Here, the notions of the locus of value generation and the focus of value creation ( Dudau *et al.* , 2019 ) may help to concretise the actors potentially involved. Thus, the further elaboration of the value (co)creation concept in an ecosystem context contributes to the public service logic and management literature. For example, the locus and focus ( Dudau *et al.,* 2019 ) of value creation needs to be expanded, i.e. the levels of individual, community, organisation and/or societal value creation. Given the experiences from NPM, ideas from the private sector need to be better adapted to the public sector context. Value generation takes place at multiple and specific levels: individual, community, organisation and/or society; this is the locus. Simultaneously, value is created *for* specific levels. For example, individuals creating value at a societal level can be classed as altruism/volunteering. Dudau *et al.* (2019) classify individuals creating value for themselves to be rational self-interest. This is no criticism, but this notion of self-interest underpins much of the goods-dominant thinking and even influences the service-marketing approaches of Vargo and Lusch (2016) , and Lovelock and Gummesson (2004) .

This integral and often unconscious bias towards viewing value creation as being inherently individualistic and dyadic breaks down when we consider the logics of value co-creation in the public sector. Here, as well as being involved in generating value, all levels may also benefit from that value, albeit sometimes in very different ways. Moreover, in public healthcare, the principle of prioritising those in greatest need entails that those in less need may have to wait, which may affect their perceptions of value.

Value, thus, is more akin to a “zero-sum game” in the public sector, whereas private-sector interactions generally see value as a more or less limitless substance. Provided that service consumers are happy to pay an amount that satisfies the service provider (and their shareholders) for a service the consumer considers sufficient for their needs and wants, value can be created bilaterally. However, value is a finite resource in many public sector contexts, particularly those with a regulatory or enforcement element. Simply put, an objection to a planning application results in a winner and a loser. Healthcare for the most acute or chronic conditions may also be unable to create value in terms of outcome (i.e. being cured) and instead must focus on value in terms of experience. Again, we reiterate that treating cancer is not like providing a haircut. Moreover, actors in a well-being ecosystem (whether service user or organisation) may not only contribute with individual/organisational value for themselves but also contribute to so-called public value; they can contribute to the “common good” or “public interest” ( Jørgensen and Bozeman, 2007 ).

Another significant aspect is user involvement, which we argue is pivotal in well-being ecosystem development and orchestration. Still, “many public sector organizations still design *for* rather than *with* service users” ( Trischler *et al.* , 2019 , p. 1614). However, the context of health and healthcare poses particular challenges to such commitments. For instance, some patients may likely be too sick to get involved to any greater extent ( Berry and Bendapudi, 2007 ). Moreover, there may be (traditional) power asymmetries between patients and, especially, physicians ( Nordgren, 2009 ). Thus, there are diverse reasons why patients and other service users may be reluctant or unwilling to get involved with staff and other actors than is the case for the common private sector “customer” ( Osborne, 2018 ).

Further, the HoP case serves as an illustrative example of a grassroots initiative of how end-users can drive innovation and realise social needs that are not met by the market or the public sector. Centering lived experience at the centre has served as the basis for a social innovation that can play a new, intermediary role in the liminal space between institutional cancer care and other social functions, and thus move the perspective from delivering healthcare to improving well-being. As Voorberg *et al.* (2015) put it, co-creation with citizens is a necessary condition to create innovative public services that truly meet the needs of those citizens, given many societal challenges.

## Conclusion

Users as active participants and collaborative partners in exchanges (e.g. Osborne, 2018 ) has received substantial attention in the public service management literature. However, research on users' involvement and multi-actor value co-creation in ecosystems to tackle complex societal challenges remains rather scarce. Based on insights from the ecosystem ( Adner, 2017 ; Ganco *et al.* , 2020 ; Jacobides *et al.* , 2018 ) value co-creation ( Galvagno and Dalli, 2014 ; Kapoor and Agarwal, 2017 ) and user involvement ( Porter and Lee, 2013 ; Osborne *et al.* , 2013 ) literature to theorise a longitudinal case study of Sweden's first support centre for people affected by cancer, we aim to contribute to this gap in knowledge. Thus, we have addressed how users' involvement in value co-creation influence the development and orchestration of well-being ecosystems to help tackle complex societal challenges. This is an important extension to the ideas contained within Public Service Logic, as it begins to address the need for systemic and rich elaborations of how public services operate.

Such an approach moves beyond the individualised focus on the user/citizen in many of the “co-concepts” and, at the same time, recognises the importance of users' experiences that is oft-neglected in-network and collaborative government models in the public sector. Consequently, value is created not only for the users/citizens participating in co-production/co-design ( Osborne and Strokosch, 2013 ) but for a multiplicity of participating actors as well as for the newly innovated product, process and/or service itself.

The paper also challenges the representativity of participating users/citizens and that, depending on their background and function, they may bring different – sometimes conflicting – knowledge and skills to the collaborative table. Drawing from an action research project ( Bradbury, 2010 ; Brydon-Miller *et al.* , 2003 ) focussing on Sweden's first support centre for people affected by cancer, we show that multi-actor resource reconfiguration provides a platform for value co-creation, innovative health services and more efficient use of resources. More specifically, the common themes include a need for multi-actor collaborations to reconfigure heterogeneous resources ( Poblete, 2021 ; Prenkert *et al.* , 2022 ); actors' adaptive change capabilities; the role of governance mechanisms to align the diverse ecosystem components, and the engagement of essential actors. We propose a framework for analysing the emergent well-being ecosystems, which shows how the efficiency of such organising structures can result in adding essential value to public services.

### Practical implications

This study has several practical implications. It demonstrates the value of cancer-affected individuals, with “lived experiences”, acting as sources for social innovation ( Alam, 2006 ; Bogers *et al.* , 2010 ; Voorberg *et al.* , 2015 ) and drivers of ecosystem development. Thus, user-driven social innovation, which takes a holistic approach to the “life event”, can operate as a platform for collaboration between different actors ( Osborne *et al.* , 2016 ). The findings also suggest that participating actors can benefit from joining the ecosystem to explore opportunities and recombine resources that contribute to the development of new products and services. Further, they imply that participating actors in the ecosystem should utilise wider knowledge and experience to tackle complex societal challenges associated with well-being.

Further, the study shows a practical application of a design thinking process inspired by the Double Diamond ( Brown, 2009 ). In this way, the findings can be helpful for other researchers who wish to move this research agenda forward. The initial workshops that brought together many stakeholders proved central to creating commitment and legitimacy within what would later become the ecosystem. Initial meetings with the “the whole system in the room” ( Huzzard *et al.* , 2017 ), set the stage for later activities that specified the shared goal. This switching between divergent (explore) and convergent (define) thinking (i.e. double diamond) in various central themes (e.g. the content of the activity, form of operation, design of the premises) during the course of the project made it possible for different people to be involved in themes where they felt they could best contribute.

### Policy implications

The findings of this study also have several policy implications. Since we demonstrate the importance of how users' involvement in value co-creation influences the orchestration of innovative, well-being ecosystems, our findings suggest policymakers should encourage the formation of ecosystems with diverse actors and resources that can help patients navigate health challenges in the broader sense. The findings especially show the potential of starting from the user's needs and life situation when the ambition is to integrate and innovate in fragmented systems.

Further, Hardyman *et al.* (2019) suggest that healthcare policy should better recognise the importance of interactions in healthcare encounters by focussing not only on outcomes of care but equally on processes of care. We agree but based on previous co-creation research in a cancer care context ( Danaher *et al.* , 2023 ; McColl-Kennedy *et al.* , 2012 ; Sweeney *et al.* , 2015 ) we would like to add that such policy should not be restricted to interactions in healthcare encounters. Indeed, our research suggests that value co-creation activities revolve around the whole patient journey in which interactions with family and friends may have the greatest positive effect on well-being rather than just interactions with healthcare staff ( McColl-Kennedy *et al.* , 2012 ).

### Limitations and future research

Despite the implications and contributions of this study, it has several limitations to which future studies should pay greater attention. The characteristics of users' involvement for value co-creation in the development and orchestration of innovative, well-being ecosystems might vary by countries. For example, the specific regulations in Sweden could facilitate, distort, or impede users' involvement for value co-creation in well-being ecosystems. Future exploration could consider specific characteristics in other countries and their impact on how users' involvement in value co-creation influences the orchestration of well-being ecosystems. Also, empirical data led to an analysis of a four-year period.

Future studies could explore longer periods. Finally, we hope future research might address three tensions: first, to find the balance between the desire for universal, generalised concepts or processes and the oftentimes detailed or “niche” focus of much extant case research. Second, future studies could address the zero-sum game/infinite value challenge faced by the public/private sector locus that we highlighted earlier. Third, we would argue that research into co-creation (and particularly in the public sector) is perhaps of reduced value unless its application can improve policy and practice rather than remaining *only* in the realms of the theoretical – we recognise this is contentious but as scholars, we feel strongly about improving public services. Finally, we make no claims for the findings of this study to be representative or generalisable in any positivist sense. Although using a case study approach has revealed stimulating insights, additional data collection, multiple cases and quantitative studies are prompted.

## Figures and Tables

**Table 1:** Background of interviewees

Respondents	Description
9 patients/relatives (referred to as “patient” in Figure 1 )	7 women (4 work, 3 retired)
	2 men (both retired)
12 organisation representatives (referred to as “organisation” in Figure 1 )	3 hospital
	2 municipality
	2 social insurance agency
	1 business-owner
	2 regional
	1 regional cancer centre (9 women, 2 men)

**Source(s):** Authors' own

**Table 2:** Needs and perspectives expressed by key actors in the three phases

*Initiating the ecosystem (Jan. 2016–April 2016)* *Main data collection method: Interviews, focus groups, design workshops*		
*Patient and Relatives Council* - cure loneliness - desire to meet others in the same situation to share experiences - get positive role models - social, emotional and practical support that complements the traditional healthcare offer	*RCC West* - a regional mandate to drive improvements in cancer care - mission to implement the national cancer strategy - create patient-focused and integrated cancer pathways - develop the psycho-social support and rehabilitation - increase the patients' position in cancer care	*Chalmers University of Technology* - expertise in innovation and change management - action researcher's knowledge and skills integrated in the innovation process - action researchers as orchestrators of innovations - systematic inquiry, documentation and analysis - integrated reflective dialogue
*Organ* *isi* *ng for value co-creation in the ecosystem (May 2016 – Dec. 2016)* *Main data collection method: focus groups, design workshops*		
*Hospital* - sees a benefit that cancer patients can meet others in similar situations - doesn't want patients to get “stuck in their disease” - sees the value of a support function outside the hospital - confidentiality policies make it difficult for staff to connect patients - healthcare focuses on the disease, not the healthy - expresses that there is a lack of time to connect with patients	*Social Insurance Agency* - aware of the health aspect of a return to work and having meaningful tasks in life - staff often feels inadequate in handling cancer patients - a cancer disease leads to lost wages and increase stress on the patient and its employer - the uncertainty of a cancer disease makes it more difficult to contribute to patients on sick leave regaining health and promoting a return to work - needs to establish good collaboration with the patient and its employer to achieve a return to working life (if possible)	*Employment Agency* - aware of the health aspect of a return to working life and having meaningful tasks - focuses primarily on the clients' workability (not the underlying disease), therefore hard to pinpoint the needs of cancer patients - wants to offer a contact person for patients and families - open for dialogue around job training and similar activities - conducts work-oriented rehabilitation - offer retraining if necessary
*Collective impact of the ecosystem (Feb. 2018 – June 2020)* *Main data collection method: interviews, focus groups*		
*Region/Hospital* - an important “bridge” between healthcare and patients - the location of HoP could be discussed, but “what's within the walls is perfect” - important that HoP addresses issues not related to healthcare, but rather to “complement” traditional healthcare responsibilities of regions/hospitals	*Municipality* - benefits when public organisations work with private actors – strengths from two sectors - HoP work together with public services provided by the municipality in the same house - improved insight of how other organisations (public, private and non-profit) work, including rules	*Local businesses* - different from selling a product or service to gain profit, but a lot of business practices can be transferred to HoP - private actors can support HoP with other things than money or furniture, e.g. knowledge and skills of running a business - a local tradition to build on of business-owners acting as “society entrepreneurs”

**Note(s):** Activities in 2017 have been excluded in the thematic timeline because the main activities were of more administrative nature

**Source(s):** Authors' own

## Note

1. Activities in 2017 have been excluded in the thematic timeline. The reason is that the majority of activities during this period were thematically of a different, more administrative, nature; registration of the association, creation of a board, renovation of premises, administration regarding financing, etc.

## References

[ref109] Adner , R ( 2006 ), “ Match your innovation strategy to your innovation ecosystem ”, Harvard Business Review , Vol. 84 No. 4 , pp. 39 - 58 . 16579417

[ref001] Adner , R. ( 2017 ), “ Ecosystem as structure: an actionable construct for strategy ”, Journal of Management , Vol. 43 , pp. 39 - 58 .

[ref002] Agranoff , R. and McGuire , M. ( 2003 ), Collaborative Public Management: New Strategies for Local Governments , Georgetown University Press , Washington, DC .

[ref003] Alam , I. ( 2006 ), “ Removing the fuzziness from the fuzzy front-end of service innovations through customer interactions ”, Industrial Marketing Management , Vol. 35 , pp. 468 - 480 .

[ref004] Alexander , V.D. , Thomas , H. , Cronin , A. , Fielding , J. and Moran-Ellis , J. ( 2008 ), Mixed Methods , Researching Social Life , Los Angeles, California , Vol. 3 , pp. 119 - 139 .

[ref005] Alexy , O. , George , G. and Salter , A. ( 2013 ), “ Cui bono? The selective revealing of knowledge and its implications for innovative activity ”, Academy of Management Review , Vol. 38 , pp. 270 - 291 .

[ref006] Alvesson , M. and Kärreman , D. ( 2007 ), “ Constructing mystery: empirical matters in theory development ”, Academy of Management Review , Vol. 32 , pp. 1265 - 1281 .

[ref007] Anker , T.B. , Sparks , L. , Moutinho , L. and Grönroos , C. ( 2015 ), “ Consumer dominant value creation: a theoretical response to the recent call for a consumer dominant logic for marketing ”, European Journal of Marketing , Vol. 49 , pp. 3 - 4 .

[ref008] Argyris , C. ( 2004 ), Reasons and Rationalizations: The Limits to Organizational Knowledge , Oxford University Press , Oxford .

[ref009] Barry , M.J. and Edgman-Levitan , S. ( 2012 ), “ Shared decision making — the pinnacle of patient-centered care ”, The New England Journal of Medicine , Vol. 366 , pp. 780 - 781 . 22375967 10.1056/NEJMp1109283

[ref010] Bell , E. , Bryman , A. and Harley , B. ( 2018 ), Business Research Methods , Oxford University Press , Oxford .

[ref011] Bergman , B. , Hellström , A. , Lifvergren , S. and Gustavsson , S.M. ( 2015 ), “ An emerging science of improvement in health care ”, Quality Engineering , Vol. 27 No. 1 , pp. 17 - 34 .

[ref012] Berry , L.L. and Bendapudi , N. ( 2007 ), “ Health care: a fertile field for service research ”, Journal of Service Research , Vol. 10 , pp. 111 - 122 .

[ref013] Berwick , D.M. ( 2009 ), “ What ‘patient-centered’ should mean: confessions of an extremist: a seasoned clinician and expert fears the loss of his humanity if he should become a patient ”, Health Affairs , Vol. 28 , pp. 555 - w565 . 10.1377/hlthaff.28.4.w555 19454528

[ref014] Bogers , M. , Afuah , A. and Bastian , B. ( 2010 ), “ Users as innovators: a review, critique, and future research directions ”, Journal of Management , Vol. 36 , pp. 857 - 875 .

[ref015] Bradbury , H. ( 2010 ), “ What is good action research? Why the resurgent interest? ”, Action Research Journal , Vol. 8 , pp. 93 - 109 .

[ref016] Brown , T. ( 2009 ), Change by Design: How Design Thinking Transforms Organizations and Inspires Innovation , Harper Business , New York .

[ref017] Brydon-Miller , M. , Greenwood , D. and Maguire , P. ( 2003 ), “ Why action research? ”, Action Research , Vol. 1 , pp. 9 - 28 .

[ref018] Bryson , J.M. , Crosby , B.C. and Seo , D. ( 2022 ), “ Strategizing on behalf of social enterprises: the case of the metropolitan economic development association and catalyst ”, Public Management Review , Vol. 24 No. 1 , pp. 124 - 141 .

[ref019] Chou , H.H. and Zolkiewski , J. ( 2012 ), “ Decoding network dynamics ”, Industrial Marketing Management , Vol. 41 , pp. 247 - 258 .

[ref020] Christensen , T. and Lægreid , P. ( 2011 ), “ Democracy and administrative policy: contrasting elements of new public management (NPM) and post-NPM ”, European Political Science Review , Vol. 3 , pp. 125 - 146 .

[ref021] Clarysse , B. , Wright , M. , Bruneel , J. and Mahajan , A. ( 2014 ), “ Creating value in ecosystems: crossing the chasm between knowledge and business ecosystems ”, Research Policy , Vol. 43 , pp. 1164 - 1176 .

[ref022] Coghlan , D. and Brannick , T. ( 2014 ), Understanding Action Research. Doing Action Research in Your Own Organization , Sage , 4th ed. , London , pp. 43 - 62 .

[ref023] Corbin , J. and Strauss , A. ( 2014 ), Basics of Qualitative Research: Techniques and Procedures for Developing Grounded Theory , Sage Publications , London .

[ref024] Danaher , T. , Danaher , P. , Sweeney , J. and McColl-Kennedy , J. ( 2023 ), “ Dynamic customer value cocreation in healthcare ”, Journal of Service Research . doi: 10.1177/10946705231161758 (in press) .

[ref025] Dattée , B. , Alexy , O. and Autio , E. ( 2018 ), “ Maneuvering in poor visibility: how firms play the ecosystem game when uncertainty is high ”, Academy of Management Journal , Vol. 61 , pp. 466 - 498 .

[ref026] Davis , J.P. ( 2016 ), “ The group dynamics of interorganizational relationships: collaborating with multiple partners in innovation ecosystems ”, Administrative Science Quarterly , Vol. 61 , pp. 621 - 661 .

[ref027] Donetto , S. , Pierri , P. , Tsianakas , V. and Robert , G. ( 2015 ), “ Experience-based co-design and healthcare improvement: realizing participatory design in the public sector ”, The Design Journal , Vol. 18 , pp. 227 - 248 .

[ref028] Dubois , A. and Gadde , L.E. ( 2002 ), “ Systematic combining: an abductive approach to case research ”, Journal of Business Research , Vol. 55 , pp. 553 - 560 .

[ref029] Dudau , A. , Glennon , R. and Verschuere , B. ( 2019 ), “ Following the yellow brick road? (Dis) enchantment with co-design, co-production and value co-creation in public services ”, Public Management Review , Vol. 21 , pp. 1577 - 1594 .

[ref030] Eisenhardt , K.M. ( 1989 ), “ Building theories from case study research ”, Academy of Management Review , Vol. 14 , pp. 532 - 550 .

[ref031] Ennis , R.D. , Hu , L. , Ryemon , S.N. , Lin , J. and Mazumdar , M. ( 2018 ), “ Brachytherapy-based radiotherapy and radical prostatectomy are associated with similar survival in high-risk localized prostate cancer ”, Journal of Clinical Oncology , Vol. 36 No. 12 , pp. 1192 - 1198 . 29489433 10.1200/JCO.2017.75.9134

[ref032] Eriksson , E. ( 2019 ), “ Representative co-production: broadening the scope of the public service logic ”, Public Management Review , Vol. 21 , pp. 291 - 314 .

[ref033] Eriksson , E. , Andersson , T. , Hellström , A. , Gadolin , C. and Lifvergren , S. ( 2020 ), “ Collaborative public management: coordinated value propositions among public service organizations ”, Public Management Review , Vol. 22 , pp. 791 - 812 .

[ref034] Eriksson , E. and Hellström , A. ( 2021 ), “ Multi-actor resource integration: a service approach in public management ”, British Journal of Management , Vol. 32 , pp. 456 - 472 .

[ref035] Fishkin , J.S. ( 2011 ), When the People Speak: Deliberative Democracy and Public Consultation , Oxford University Press , Oxford .

[ref036] Frow , P. , Nenonen , S. , Payne , A. and Storbacka , K. ( 2015 ), “ Managing Co-creation design ”, British Journal of Management , Vol. 26 , pp. 463 - 483 .

[ref037] Fusch , P.I. and Ness , L.R. ( 2015 ), “ Are we there yet? Data saturation in qualitative research ”, The Qualitative Report , Vol. 20 No. 9 , p. 1408 .

[ref038] Galunic , D.C. and Rodan , S. ( 1998 ), “ Resource recombinations in the firm: knowledge structures and the potential for Schumpeterian innovation ”, Strategic Management Journal , Vol. 19 , pp. 1193 - 1201 .

[ref039] Galvagno , M. and Dalli , D. ( 2014 ), “ Theory of value co-creation: a systematic literature review ”, Managing Service Quality , Vol. 24 , pp. 643 - 683 .

[ref040] Ganco , M. , Kapoor , R. and Lee , G.K. ( 2020 ), “ From rugged landscapes to rugged ecosystems: structure of interdependencies and firms' innovative search ”, Academy of Management Review , Vol. 45 , pp. 646 - 674 .

[ref041] Gioia , D.A. , Corley , K.G. and Hamilton , A.L. ( 2013 ), “ Seeking qualitative rigor in inductive research: notes on the Gioia methodology ”, Organizational Research Methods , Vol. 16 , pp. 15 - 31 .

[ref042] Golafshani , N. ( 2003 ), “ Understanding reliability and validity in qualitative research ”, The Qualitative Report , Vol. 8 , pp. 597 - 607 .

[ref043] Grönroos , C. ( 2011 ), “ Value co-creation in service logic: a critical analysis ”, Marketing Theory , Vol. 11 , pp. 279 - 301 .

[ref044] Grönroos , C. ( 2019 ), “ Reforming public services: does service logic have anything to offer? ”, Public Management Review , Vol. 21 , pp. 775 - 788 .

[ref045] Gruner , K.E. and Homburg , C. ( 2000 ), “ Does customer interaction enhance new product success? ”, Journal of Business Research , Vol. 49 , pp. 1 - 14 .

[ref046] Hardyman , W. , Daunt , K.L. and Kitchener , K. ( 2015 ), “ Value co-creation through patient engagement in health care: a micro-level approach and research agenda ”, Public Management Review , Vol. 17 , pp. 90 - 107 .

[ref047] Hardyman , W. , Kitchener , M. and Daunt , K.L. ( 2019 ), “ What matters to me! User conceptions of value in specialist cancer care ”, Public Management Review , Vol. 21 , pp. 1687 - 1706 .

[ref048] Hendriks , C.M. ( 2012 ), The Politics of Public Deliberation: Citizen Engagement and Interest Advocacy , Springer , New York .

[ref049] Huzzard , T. , Hellström , A. and Lifvergren , S. ( 2017 ), “ Whole system in the room: toward systems integration in healthcare ”, Health Communication , Vol. 33 , pp. 800 - 808 . 28467191 10.1080/10410236.2017.1314854

[ref050] Jacobides , M.G. , Cennamo , C. and Gawer , A. ( 2018 ), “ Towards a theory of ecosystems ”, Strategic Management Journal , Vol. 39 , pp. 2255 - 2276 .

[ref051] Jørgensen , T.B. and Bozeman , B. ( 2007 ), “ Public values: an inventory ”, Administration and Society , Vol. 39 , pp. 354 - 381 .

[ref052] Kapoor , R. ( 2018 ), “ Ecosystems: broadening the locus of value creation ”, Journal of Organization Design , Vol. 7 , p. 12 .

[ref053] Kapoor , R. and Agarwal , S. ( 2017 ), “ Sustaining superior performance in business ecosystems: evidence from application software developers in the iOS and Android smartphone ecosystems ”, Organization Science , Vol. 28 , pp. 531 - 551 .

[ref054] Kapoor , R. and Lee , J.M. ( 2013 ), “ Coordinating and competing in ecosystems: how organizational forms shape new technology investments ”, Strategic Management Journal , Vol. 34 , pp. 274 - 296 .

[ref055] Karim , S. ( 2006 ), “ Modularity in organizational structure: the reconfiguration of internally developed and acquired business units ”, Strategic Management Journal , Vol. 27 , pp. 799 - 823 .

[ref056] Kinder , T. , Six , F. , Stenvall , J. and Memon , A. ( 2022 ), “ Governance-as-legitimacy: are ecosystems replacing networks? ”, Public Management Review , Vol. 24 No. 1 , pp. 8 - 33 .

[ref057] Langley , A.N. , Smallman , C. , Tsoukas , H. and Van de Ven , A.H. ( 2013 ), “ Process studies of change in organization and management: unveiling temporality, activity, and flow ”, Academy of Management Journal , Vol. 56 , pp. 1 - 13 .

[ref058] Lifvergren , S. , Huzzard , T. and Hellström , A. ( 2015 ), “ Action research and healthcare ”, Action Research’ , Vol. 13 , pp. 3 - 8 .

[ref059] Lincoln , Y. and Guba , E. ( 1985 ), Naturalistic Inquiry , Sage , Newbury Park .

[ref060] Locke , K. , Golden-Biddle , K. and Feldman , M.S. ( 2008 ), “ Perspective—making doubt generative: rethinking the role of doubt in the research process ”, Organization Science , Vol. 19 , pp. 907 - 918 .

[ref061] Lovelock , C. and Gummesson , E. ( 2004 ), “ Whither services marketing? In search of a new paradigm and fresh perspectives ”, Journal of Service Research , Vol. 7 , pp. 20 - 41 .

[ref062] McColl-Kennedy , J.R. , Vargo , S.L. , Dagger , T.S. , Sweeney , J.C. and Kasteren , Y.V. ( 2012 ), “ Health care customer value cocreation practice styles ”, Journal of Service Research , Vol. 15 , pp. 370 - 389 .

[ref063] Michie , S. , Miles , J. and Weinman , J. ( 2003 ), “ Patient-centredness in chronic illness: what is it and does it matter? ”, Patient Education and Counseling , Vol. 51 , pp. 197 - 206 . 14630376 10.1016/s0738-3991(02)00194-5

[ref064] Mintzberg , H. ( 2017 ), Managing the Myths of Health Care: Bridging the Separations between Care, Cure, Control, and Community , Berrett-Koehler , Oakland .

[ref065] Mitchell , R.K. , Agle , B.R. and Wood , D.J. ( 1997 ), “ Toward a theory of stakeholder identification and salience: defining the principle of who and what really counts ”, Academy of Management Review , Vol. 22 , pp. 853 - 886 .

[ref066] Moore , J.F. ( 2006 ), “ Business ecosystems and the view from the firm ”, The Antitrust Bulletin , Vol. 51 , pp. 31 - 75 .

[ref067] Nenonen , S. and Storbacka , K. ( 2010 ), “ Business model design: conceptualizing networked value co-creation ”, International Journal of Quality and Service Sciences , Vol. 2 , pp. 43 - 59 .

[ref068] Nordgren , L. ( 2009 ), “ Value creation in health care services–developing service productivity: experiences from Sweden ”, International Journal of Public Sector Management , Vol. 22 , pp. 114 - 127 .

[ref069] Olsson , E.M. ( 2016 ), “ Interpersonal complaints regarding cancer care through a gender lens ”, International Journal for Health Care Quality Assurance , Vol. 29 , pp. 687 - 702 . 10.1108/IJHCQA-03-2014-0032 27298065

[ref070] Osborne , S. ( 2018 ), “ From public service-dominant logic to public service logic: are public service organizations capable of co-production and value co-creation? ”, Public Management Review , Vol. 20 , pp. 225 - 231 .

[ref071] Osborne , S.P. ( 2020 ), Public Service Logic: Creating Value for Public Service Users, Citizens, and Society through Public Service Delivery , Routledge , Abington .

[ref072] Osborne , S. and Strokosch , K. ( 2013 ), “ It takes two to tango? Understanding the Co-production of public services by integrating the services management and public administration perspectives ”, British Journal of Management , Vol. 24 , pp. S31 - S47 .

[ref073] Osborne , S. , Radnor , Z. and Nasi , G. ( 2013 ), “ A new theory for public service management? Toward a (public) service-dominant approach ”, American Review of Public Administration , Vol. 43 , pp. 135 - 158 .

[ref074] Osborne , S.P. , Radnor , Z. and Strokosch , K. ( 2016 ), “ Co-production and the co-creation of value in public services: a suitable case for treatment? ”, Public Management Review , Vol. 18 , pp. 639 - 653 .

[ref075] Osborne , S.P. , Powell , M. , Cui , T. and Strokosch , K. ( 2021 ), “ New development:‘Appreciate–Engage–Facilitate’—the role of public managers in value creation in public service ecosystems ”, Public Money and Management , Vol. 41 No. 8 , pp. 668 - 671 .

[ref076] Osborne , S.P. , Powell , M. , Cui , T. and Strokosch , K. ( 2022 ), “ Value creation in the public service ecosystem: an integrative framework ”, Public Administration Review , Vol. 82 No. 4 , pp. 634 - 645 .

[ref077] Osterwalder , A. and Pigneur , Y. ( 2010 ), Business Model Generation: A Handbook for Visionaries, Game Changers, and Challengers , Routledge , Chichester , Vol. 1 .

[ref078] Petrescu , M. ( 2019 ), “ From marketing to public value: towards a theory of public service ecosystems ”, Public Management Review , Vol. 21 , pp. 1733 - 1752 .

[ref079] Poblete , L. ( 2021 ), “ Resource transformation in the reconstitution of broken interorganizational relationships ”, Journal of Strategy and Management , Vol. 14 No. 2 , pp. 207 - 226 .

[ref080] Poblete , L. , Kadefors , A. , Rådberg , K.K. and Gluch , P. ( 2022 ), “ Temporality, temporariness and keystone actor capabilities in innovation ecosystems ”, Industrial Marketing Management , Vol. 102 , pp. 301 - 310 .

[ref081] Pollitt , C. and Bouckaert , G. ( 2017 ), Public Management Reform: A Comparative Analysis – into the Age of Austerity , Oxford University Press , Oxford .

[ref082] Porter , M.E. and Lee , T.H. ( 2013 ), The Strategy that Will Fix Health Care , Harvard Business Review , Vol. 91 , p. 24 .

[ref083] Porter , M. and Teisberg , E. ( 2006 ), Redefining Health Care: Creating Value-Based Competition on Results , Harvard Business School Press , Boston .

[ref084] Prahalad , C.K. and Ramaswamy , V. ( 2004 ), “ Co-creation experiences: the next practice in value creation ”, Journal of Interactive Marketing , Vol. 18 , pp. 5 - 14 .

[ref085] Prenkert , F. , Hedvall , K. , Hasche , N. , Frick , J.E. , Abrahamsen , M.H. , Aramo-Immonen , H. , Baraldi , E. , Bocconcelli , R. , Harrison , D. , Huang , L. and Huemer , L. ( 2022 ), “ Resource interaction: key concepts, relations and representations ”, Industrial Marketing Management , Vol. 105 , pp. 48 - 59 .

[ref086] Provan , K.G. and Kenis , P. ( 2008 ), “ Modes of network governance: structure, management, and effectiveness ”, Journal of Public Administration Research and Theory , Vol. 18 , pp. 229 - 252 .

[ref087] Ruijer , E. , Dingelstad , J. and Meijer , A. ( 2023 ), “ Studying complex systems through design interventions probing open government data ecosystems in The Netherlands ”, Public Management Review , Vol. 25 No. 1 , pp. 129 - 149 .

[ref088] Siggelkow , N. ( 2007 ), “ Persuasion with case studies ”, Academy of Management Journal , Vol. 50 , pp. 20 - 24 .

[ref089] Silverman , D. ( 2015 ), Interpreting Qualitative Data , Sage , London .

[ref090] Sinkovics , R.R. and Alfoldi , E.A. ( 2012 ), “ Progressive focusing and trustworthiness in qualitative research ”, Management International Review , Vol. 52 No. 6 , pp. 817 - 845 .

[ref091] Sjövall , K. , Gunnars , B. , Olsson , H. and Thomé , B. ( 2011 ), “ Experiences of living with advanced colorectal cancer from two perspectives–inside and outside ”, European Journal of Oncology Nursing , Vol. 15 No. 5 , pp. 390 - 397 . 21163701 10.1016/j.ejon.2010.11.004

[ref092] Skålén , P. , Karlsson , J. , Engen , M. and Magnusson , P.R. ( 2018 ), “ Understanding public service innovation as resource integration and creation of value propositions ”, Australian Journal of Public Administration , Vol. 77 No. 4 , pp. 700 - 714 .

[ref093] Smith , F. , Hellström , A. , Gunnarsdóttir , K.Á. , Genell , A. , Eriksson , E. , Mannefred , C. , Björk-Eriksson , T. and Vaughn , L. ( 2021 ), “ Exploring the meaning, role and experiences of a patient-led social innovation for people affected by cancer: a new collaborative care model complementing traditional cancer rehabilitation in Sweden ”, BMJ Open Quality , Vol. 10 No. 4 , p. e001400 . 10.1136/bmjoq-2021-001400 PMC8543647 34686486

[ref094] Socialstyrelsen and Cancerfonden ( 2013 ), Cancer I Siffror 2013 (Cancer by Numbers 2013) , Socialstyrelsen , Stockholm , ISBN: 978-91-89446-64-9 .

[ref095] Sweeney , J. , Danaher , T. and McColl-Kennedy , J. ( 2015 ), “ Customer effort in value cocreation activities improving quality of life and behavioral intentions of health care customers ”, Journal of Service Research , Vol. 18 , pp. 318 - 335 .

[ref096] Tranfield , D. , Denyer , D. and Smart , P. ( 2003 ), “ Towards a methodology for developing evidence‐informed management knowledge by means of systematic review ”, British Journal of Management , Vol. 14 , pp. 207 - 222 .

[ref097] Trischler , J. , Dietrich , T. and Rundle-Thiele , S. ( 2019 ), “ Co-design: from expert-to user-driven ideas in public service design ”, Public Management Review , Vol. 21 No. 11 , pp. 1595 - 1619 .

[ref110] Trischler , J , Pervan , S.J , Kelly , S.J and Scott , D.R ( 2018 ), “ The value of codesign: The effect of customer involvement in service design teams ”, Journal of Service Research , Vol. 21 No. 1 , pp. 75 - 10 .

[ref098] Tronvoll , B. ( 2012 ), “ A dynamic model of customer complaining behaviour from the perspective of service‐dominant logic ”, European Journal of Marketing , Vol. 46 , pp. 284 - 305 .

[ref099] Vargo , S. and Akaka , M. ( 2012 ), “ Value cocreation and service systems (re) formation: a service ecosystems view ”, Service Science , Vol. 4 , pp. 207 - 217 .

[ref100] Vargo , S.L. and Lusch , R. ( 2004 ), “ Evolving to a new dominant logic for marketing ”, Journal of Marketing , Vol. 68 , pp. 1 - 17 .

[ref101] Vargo , S. and Lusch , R. ( 2008 ), “ Service-dominant logic: continuing the evolution ”, Journal of the Academy of Marketing Science , Vol. 36 , pp. 1 - 10 .

[ref102] Vargo , S. and Lusch , R. ( 2016 ), “ Institutions and axioms: an extension and update of service-dominant logic ”, Journal of the Academy of Marketing Science , Vol. 44 , pp. 5 - 23 .

[ref103] Vargo , S.L. , Maglio , P.P. and Akaka , M.A. ( 2008 ), “ On value and value co-creation: a service systems and service logic perspective ”, European Management Journal , Vol. 26 No. 3 , pp. 145 - 152 .

[ref104] Von Hippel , E. ( 1986 ), “ Lead users: a source of novel product concepts ”, Management Science , Vol. 32 No. 7 , pp. 791 - 805 .

[ref105] Voorberg , W.H. , Bekkers , V.J. and Tummers , L.G. ( 2015 ), “ A systematic review of co-creation and co-production: embarking on the social innovation journey ”, Public Management Review , Vol. 17 No. 9 , pp. 1333 - 1357 .

[ref106] Yin , R.K. ( 2018 ), Case Study Research and Applications Design and Methods , 6th ed. , CA Sage , Thousand Oaks .

[ref107] Zeithaml , V.A. , Jaworski , B.J. , Kohli , A.K. , Tuli , K.R. , Ulaga , W. and Zaltman , G. ( 2020 ), “ A theories-in-use approach to building marketing theory ”, Journal of Marketing , Vol. 84 No. 1 , pp. 32 - 51 .

